# Contributions to Management Strategies in the NE Atlantic Regarding the Life History and Population Structure of a Key Deep-Sea Fish (*Mora Moro*)

**DOI:** 10.3390/biology10060522

**Published:** 2021-06-11

**Authors:** Régis Santos, Wendell Medeiros-Leal, Osman Crespo, Ana Novoa-Pabon, Mário Pinho

**Affiliations:** 1 IMAR Institute of Marine Research, University of the Azores, 9901-862 Horta, Portugal; wendell.mm.silva@uac.pt (W.M.-L.); mario.rr.pinho@uac.pt (M.P.); 2 Okeanos R&D Centre, University of the Azores, 9901-862 Horta, Portugal; osman.crespo@gmail.com (O.C.); 19anita89@gmail.com (A.N.-P.); 3 Department Oceanography and Fisheries, Faculty of Science and Technology, University of the Azores, 9901-862 Horta, Portugal

**Keywords:** common mora, demersal, commercial fish, stock structure, assessment, fisheries management, Azores

## Abstract

**Simple Summary:**

The growing scarcity of continental shelf and epipelagic oceanic fishes has led to commercial fishing in deeper waters. With this spatial expansion in fishing efforts, some vulnerable deep-sea species have been increasingly captured. To reduce fishing-induced impacts on these resources, information on population traits is required by fishery scientists to produce adequate management advice. In the Northeast Atlantic, the common mora *Mora moro* has become the main fish species caught by bottom longliners operating in deep waters between 600 and 1200 m. Information about the biology and exploitation status of this species is scarce. This study unravels and highlights important and crucial aspects of the habitat preferences, life-history traits (sex ratio, timing of reproduction, size at maturity, growth pattern, and mortality rates), size structure, and abundance of the *M. moro* based on scientific surveys and commercial fisheries in the Azores region. Results highlight its vulnerability to overfishing due to its large size, slow growth, low natural mortality, long life span, and late maturity.

**Abstract:**

With the commercial fishery expansion to deeper waters, some vulnerable deep-sea species have been increasingly captured. To reduce the fishing impacts on these species, exploitation and management must be based on detailed and precise information about their biology. The common mora *Mora moro* has become the main deep-sea species caught by longliners in the Northeast Atlantic at depths between 600 and 1200 m. In the Azores, landings have more than doubled from the early 2000s to recent years. Despite its growing importance, its life history and population structure are poorly understood, and the current stock status has not been assessed. To better determine its distribution, biology, and long-term changes in abundance and size composition, this study analyzed a fishery-dependent and survey time series from the Azores. *M. moro* was found on mud and rock bottoms at depths below 300 m. A larger–deeper trend was observed, and females were larger and more abundant than males. The reproductive season took place from August to February. Abundance indices and mean sizes in the catch were marked by changes in fishing fleet operational behavior. *M. moro* is considered vulnerable to overfishing because it exhibits a long life span, a large size, slow growth, and a low natural mortality.

## 1. Introduction

Fishing is the most widespread human exploitative activity in marine ecosystems [1]. Global fisheries produce more than 90 million tons of fish per year, providing the world’s growing population with a crucial source of food [2]. Due to the nutritional characteristics of fish, fisheries go beyond simply providing a source of calories and protein. They provide essential micronutrients and omega-3 fatty acids, which are necessary to end malnutrition and improve health around the world [3]. While the human population and global demand for fish are increasing, the growing scarcity of continental shelf and epipelagic oceanic fishes has led to commercial fishing in deeper waters [4]. 

Among the deep-sea species that became commercially exploitable with the spatial expansion of fishing efforts, the common mora *Mora moro* (Risso, 1810) has dominated longline catches in the Northeast Atlantic between 600 and 1200 m depths [5]. *Mora moro* is a bathypelagic species mainly recorded from the outer continental shelf and slope at depths between 300 and 2500 m [6,7]. It is distributed in the NE Atlantic (Iceland and Faeroes to West Africa, including the Azores and Madeira archipelagos), the Western Mediterranean Sea, the Western Indian Ocean, and the Pacific Ocean (Australia, New Zealand, and between Valparaiso, Chile, and the Juan Fernandez Islands) [6,8].

**Figure 1:**
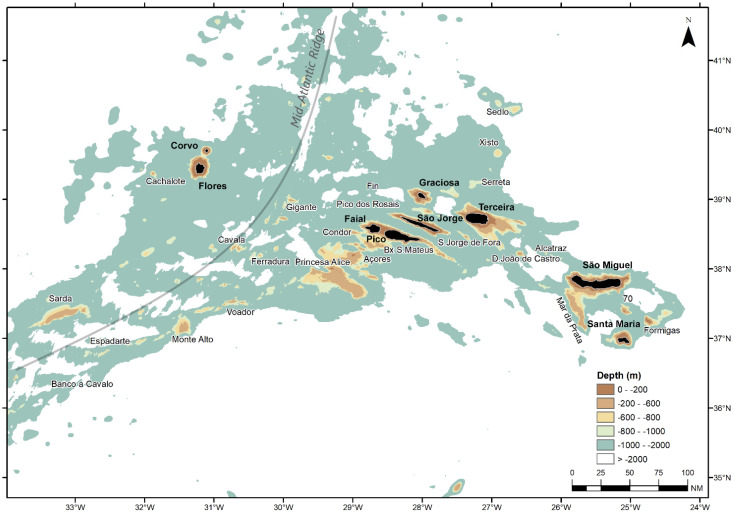
Location of the Azores archipelago (NE Atlantic Ocean), with the names of the nine islands (Corvo, Flores, Faial, Pico, São Jorge, Graciosa, Terceira, São Miguel, and Santa Maria) and main banks and seamounts. The approximate location of the Mid-Atlantic Ridge is shown as a light gray line.

The Azores is a deep-sea and open-ocean region formed by nine volcanic islands and several seamounts (Figure 1). The Azores Exclusive Economic Zone (EEZ; ICES Subarea 10a2) is about 1 Mkm^2^ and has an average depth of 3000 m. The traditionally exploitable fishing area (i.e., habitats with depths less than 700 m) is small and covers only about 1% of the entire EEZ [9]. As management measures to reduce fishing impacts over these habitats and resources, fishing area restrictions [10,11] and the following spatial expansion of bottom longline fishing efforts to deep offshore areas have taken place over the last several decades. As a result, commercial landings of *M. moro* increased from approximately 70 t in the early 2000s to over 150 t in recent years [5].

*Mora moro* is caught in the Azores by a small-scale (fishing vessels with an overall length of less than 12 m) demersal fishery using hook and line gear in deep waters [12]. Discarding for this species was averaged at less than 5% [13] and is therefore considered to be negligible [14]. *Mora moro* catches are still at low levels when compared with other demersal species [12], but it has been increasingly considered by fishers a supplementary or alternative resource to traditionally exploited fish species [7]. Despite its growing economic importance, the life history and population structure of *M. moro* inhabiting the NE Atlantic are still poorly understood, and exploitation is not based on detailed and precise information about its biology. For satisfactory fishery management and species conservation, this type of information is essential to evaluate changes in stock in response to fishing and to predict conditions as far into the future as possible [15].

Ensuring the sustainable exploitation of fishery resources over the extent of their spatial distribution is the major goal of fishery management [16]. Control measures can be applied at several geographic scales, ranging from a large marine ecosystem (LME) to a fishing community (a cluster of villages) [17]. However, to improve efficiency and success, managers need to define the fishery management units (FMUs) they are going to work with. The proposed FMUs should include the biological processes of the species (i.e., much of the life cycle should occur within the FMU), which are needed to link the biological self-reproduction of the resources with management actions [16,18]. 

In the Azores, the available information is not sufficient to define whether caught individuals can be considered a discrete FMU [7]. At the same time, detailed data on the catch, effort, length, weight, sex, and maturity from targeted surveys in the Azorean waters, complemented by fishery data, have been collected approximately over the past 20 years [7]. Some results have been reported and discussed concerning age and growth estimations [19]. However, other fundamental biological aspects (e.g., size at maturity and mortality rates), distributional patterns, and fishing exploitation status remain unanalyzed and poorly known. 

In this context, this study aims to analyze detailed information on habitat preferences, life-history traits (sex ratio, timing of reproduction, size at maturity, growth pattern, and mortality rates), size structure, and abundance of the common mora *M. moro* derived from surveys and commercial fisheries in the Azores region. This is expected to improve input information for stock assessment and fish conservation and allow for investigations of long-term changes in abundance and size composition. The null hypothesis that the stock of this species in the Azores EEZ represents a discrete FMU was also tested by comparing the observed population structure with those from nearby areas. 

## 2. Material and Methods

### 2.1. Datasets

Three types of datasets from the Azores were analyzed in this study: survey-derived data, commercial catches, and official landings.

Bottom longline surveys were conducted each spring from 1996 to 2019 following a stratified random design that covered waters surrounding the islands and major seamounts of the Azores archipelago. Each sampling area was divided into depth strata with 50 m intervals down to a 1200 m depth. Each fishing set was laid perpendicular to the depth contours. Relative abundance indices (RPN; ind. 10^−3^ hooks) were estimated as the mean catch per unit of effort weighted by the corresponding area size. Fork length (*L_F_*), weight, and sex were recorded for each fish. Detailed information on survey design and abundance index estimation procedures is given by Pinho et al. [20].

Commercial catch information was collected within the European Commission’s data collection framework (DCF) [21]. Structured interviews (*n* = 31616) were conducted with the vessels’ captains of the local fleet during the landings for the period 1990–2017. Each record included the vessel ID and detailed information on fishing operations, including the number of days at sea, the gear characteristics, the fishing locations on a pre-defined spatial grid of 10 × 10 NM, and the catch in weight for each captured species. Biological information (*L_F_*, sex, maturity stage, and gonadosomatic and hepatosomatic indices) was obtained for 172 fish specimens between 2005 and 2017. Commercial landing data (in tons) were obtained from the Azores Auction Services (Lotaçor S.A.) for the period 1990–2020. These data also included information on the *L_F_* for each fish sample (*n* = 12,405). DCF sampling design and protocols followed the outcomes of the International Council for the Exploration of the Sea working groups on commercial catches (WGCATCH; https://www.ices.dk/community/groups/Pages/WGCATCH.aspx; accessed on 5 March 2021) and biological parameters (WGBIOP; https://www.ices.dk/community/groups/Pages/WGBIOP.aspx; accessed on 5 March 2021) [22]. 

### 2.2. Data Analyses

Generalized additive models (GAMs) were used to describe relationships between presence–absence and survey-derived abundance indices (RPN) of *M. moro* and environmental predictors. A GAM uses smooth functions to fit responses to explanatory variables [23,24]. Predictor variables included in the analyses were latitude and longitude (as an interaction term), depth, and substrate type. Latitude and longitude as well as absolute depth (0–1200 m) were obtained during gear deployment in the surveys. The substrate type was extracted from EMODnet seabed habitat compilations (www.emodnet-seabedhabitats.eu, 5 March 2021) and categorized as coarse sediment (C.Sed), mixed sediment (Mix.Sed), mud (Mud), muddy sand (Mud.S), rock (Rock), sand (Sand), or sandy mud (Sand.M). A hurdle (delta) GAM approach [25,26] was applied due to the presence of a large proportion of zero values in the RPN data (80%). This approach consisted of fitting separately the presence–absence data using a binomial error distribution with a logit link function from the abundance given the presence using a Gaussian error distribution with an identity link function. Deviance results were used to identify the environmental predictor variables that explained most of the variability in the RPN data. GAM analyses were performed using the *mgcv* package [27,28,29,30,31] in R, version 4.0.3 [32].

A two-sample Kolmogorov–Smirnov (K-S) test was used to evaluate whether size–frequency distributions observed in different regions (seamount and island) during the survey or coming from different databases (survey and commercial landings) had the same statistical distribution. Differences in mean *L_F_* among years and depth strata were determined by Welch’s heteroscedastic F test and Bonferroni post-hoc correction, using the *onewaytests* R package [33] and assuming unequal variance between samples. 

Growth parameters were estimated from the *L_F_*–frequency data (1-cm class interval) taken monthly from the commercial landings for the period 2010–2016, using the von Bertalanffy growth function (VBGF). As the *L_F_* data were not separated for males and females, growth parameters were estimated for combined sexes. The VBGF analysis was modified from the original model [34] to remove theoretical age at length zero (*t*_0_), as follows:

*L_t_* = *L_∞_* (1 − e ^–*k*(*t*)^)

where *L_t_* is the *L_F_* (cm) at age *t* (year), *L_∞_* is the asymptotic *L_F_* (cm), and *k* is the growth rate coefficient (year^−1^). The asymptotic length (*L_∞_*) and growth coefficient (*k*) were calculated by electronical length frequency analysis using a bootstrapped method with a genetic algorithm (ELEFAN_GA_boot; [35]) within the *TropFishR* R package [36,37]. In this study, 1000 resamples were conducted for the bootstrap experiments. The estimated *L_∞_* and *k* were used to calculate the growth performance index (*⏀*) proposed by [38] as *⏀* = log(*k*) + 2 log(*L_∞_*). 

The proportion of males relative to females (M:F) were estimated by *L_F_*–class and depth stratum. The chi-square test was used to determine if proportions deviated significantly from 1:1. Maturity stages were classified for both sexes into six phases (0—immature, I—resting, II—developing–beginning and the development of maturation, III—Pre-spawning, IV—spawning–running, and V—spent) adapted from [39] and based on the macroscopic observation of the gonads. Maturity stages I, III, IV, and V were considered sexually mature. The length at which 50% of the individuals are mature (*L_50_*) was estimated by logistic regression (Bayes) using the *sizeMat* R package [40]. The *L_50_* was estimated only for combined sexes because of the limited sample size. The logistic function was expressed in the following way:

*P* = 1/(1 + e ^–(*β0* + *β1 X*)^)

where *P* is the probability of an individual being mature at a determinate *X* length, and *β0* (intercept) and *β1* (slope) are the parameters estimated.

The spawning season was identified from the monthly incidence of changes in the gonadosomatic index (GSI = gonad weight/total weight × 100), the hepatosomatic index (HSI = liver weight/total weight × 100), and maturity stage frequency. Significant differences in GSI and HSI throughout the year were tested using Welch’s test and Bonferroni post-hoc correction.

Mortality rates were estimated using the *L_F_*–composition data taken from the commercial fishery for the period 2010–2016. The total mortality rate (*Z*; year^−1^) was estimated using the mean length data in the non-equilibrium situations method [41]. Catchability coefficient (*q*), natural mortality (*M*), and fishing mortality (*F*) rates (year^−1^) were calculated based on the method by Then et al. [42] using additional information about fishing efforts for the period 2009–2016. The exploitation rate (*E*) was determined by *E* = *F*/(*F + M*) [43]. An *E* value close to 0.5 was considered an optimal level of exploitation, whereas *E* > 0.5 referred to an overexploitation situation [43]. The length at which 100% of individuals are vulnerable to capture (*L_c_*) was determined by using the peak of the length–frequency distribution [41].

Differences in RPN over the years were determined by Welch’s test and Bonferroni post-hoc correction. The unbiased (standardized) annual trend of the catch per unit effort (CPUE; kg days at sea^−1^ vessel^−1^) derived from the commercial fleet was estimated based on the generalized linear modeling approach using a hurdle–lognormal model [25,44,45]. Year, quarter, vessel size, fishing gear, the average depth zone of the fishing operation, and the percentage of capture of the common mora species in relation to the total (target effect) were considered potential drivers of CPUE. Standardized CPUE was estimated using the *lsmeans* R package [46]. Detailed statistical information is given by ICES WGDEEP [14].

Fishing effort was calculated as the number of days at sea per vessel based on the departure and arrival dates provided in the DCF structured interviews. Since absolute effort per fishing location was unknown, a proxy was obtained by dividing the number of days at sea per trip by the number of 10 × 10 NM grid squares fished per trip. The spatial distribution of fishing efforts was then plotted using the *marmap* R package [47]. 

Significance levels of all statistical analyses were set at a *p*-value of < 0.05.

## 3. Results

### 3.1. Habitat Preferences

**Figure 2:**
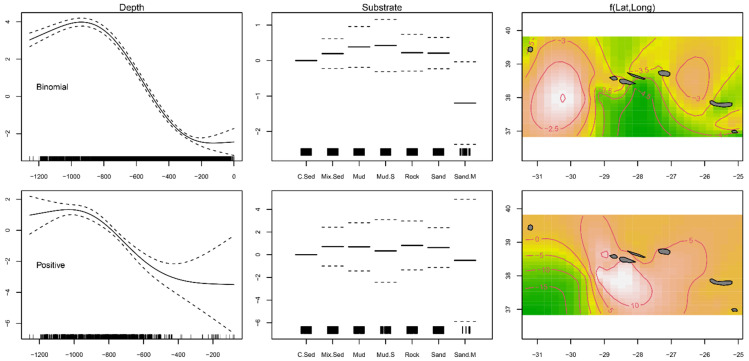
Residual plots for predictors obtained by the presence–absence binomial and abundance Gaussian generalized additive models (GAMs) for *Mora moro* caught during the surveys (1996–2019) in the Azores. Each column shows a predictor (depth, substrate and latitude and longitude) in the context of the model. Solid and dashed lines represent the smoother fit and the approximate ± 0.95 confidence interval, respectively. Observed data points are indicated as tick marks on the *x*-axis. In the 2D smoother colored plot, white color indicates more individuals and green color fewer.

The GAMs, performed to examine the joint effects of substrate type, depth, and latitude and longitude on fish distribution, indicated that the presence–absence (binomial) model explained 45.4% of the variance, while the positive catches (Gaussian) model explained 23.9% (Table 1). Substrate type was not significant for most of the levels under analysis (Table 1). The modeled data predicted a significantly lower presence of *Mora moro* on coarse sediment (*p* < 0.001) and sandy mud habitats (*p* < 0.04; Table 1; Figure 2). Abundance given the presence was lower in coarse sediment bottoms (*p* < 0.001; Table 1; Figure 2). Depth as well as latitude and longitude were indicated to have a smoothing term significantly different from zero (*p* < 0.001) in the fish presence and abundance, being, therefore, relevant variables to the model’s fit (Table 1). The curve fitted to the modeled distribution found the greatest occurrence and highest abundance in the depth range of 800–1000 m, and in sites located around the Mid-Atlantic Ridge (MAR) and in the south of the central islands group, respectively (Figure 2). 

### 3.2. Size Structure

**Figure 3:**
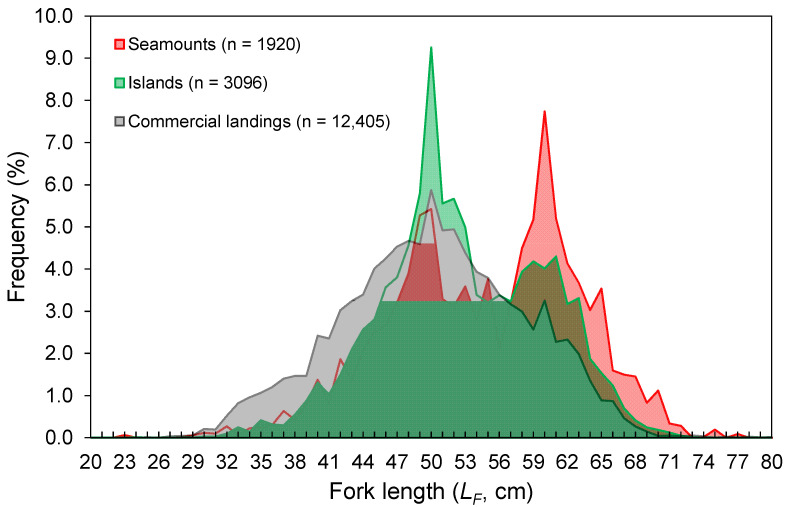
Fork length (*L_F_*)–frequency distribution of *Mora moro* derived from the surveys (1996–2019) and commercial landings (1990–2017) in the Azores. Data from surveys are shown by area (seamounts and islands) and the total number of individuals (n) refers to the total RPN (ind. 10^−3^ hooks).

**Figure 4:**
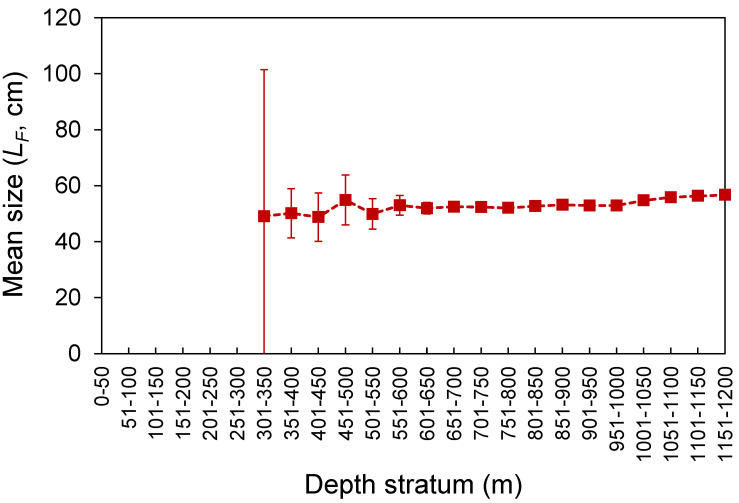
Mean (±0.95 confidence interval) fork length (*L_F_*) by depth stratum of *Mora moro* derived from the surveys (1996–2019) in the Azores.

**Figure 5:**
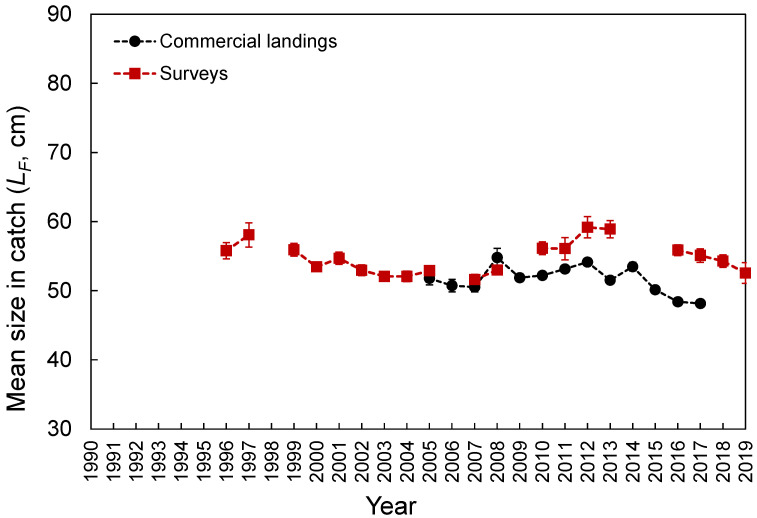
Annual Mean (±0.95 confidence interval) fork length (*L_F_*) of *Mora moro* derived from the surveys and commercial landings in the Azores.

The *L_F_* ranged from 23 to 80 cm (Figure 3). Despite the differences in the temporal coverage of the samples, the *L_F_* composition from the commercial landings was statistically similar to that observed from the spring surveys (K-S test, D = 0.065, *p* = 0.997; Figure 3). However, the *L_F_*–frequency of larger individuals was visually higher in the survey samples (Figure 3). No differences were found between seamounts and islands (K-S test, D = 0.091, *p* = 0.908; Figure 3). Larger individuals were significantly more abundant deeper than 1000 m (Welch’s test, *F* = 32.5, *p* < 0.001; Bonferroni, *p* < 0.024; Figure 4). No *L_F_*-related information by area or depth was available or was reported in the fishery-dependent dataset. The survey-derived mean *L_F_* showed significant variability among years (Welch’s test, *F* = 19.1, *p* < 0.001), with smaller individuals caught between 2002 and 2008 and larger ones between 2010 and 2016 (Bonferroni, *p* < 0.040; Figure 5). From the commercial landings, the mean *L_F_* also showed significant interannual differences (Welch’s test, *F* = 469.1, *p* < 0.001), with larger fishes in 2008, 2011, 2012, and 2014 and smaller ones between 2015 and 2017 (Bonferroni, *p* < 0.044; Figure 5).

### 3.3. Growth Parameters

The best set of growth parameters obtained from *L_F_*–frequency data for the period 2010–2016 were *L_∞_* = 77.7 cm *L_F_*, *k* = 0.07 year^−1^, and *⏀* = 2.63. Table 2 and Figure S1 present detailed information and related estimated parameters.

### 3.4. Sex Ratio

**Figure 6:**
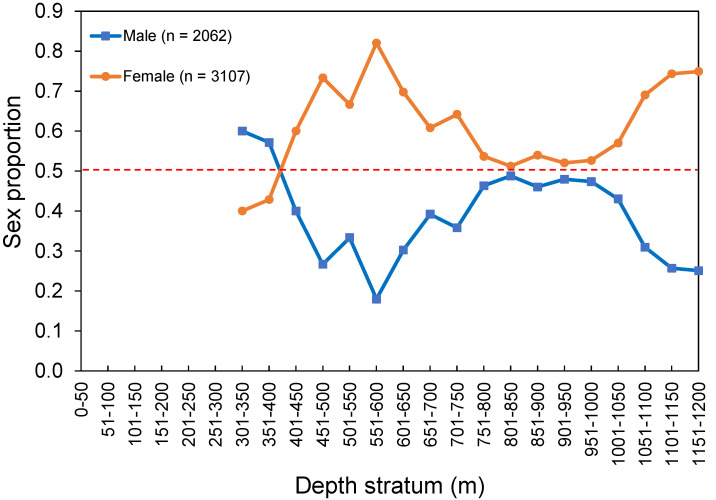
Sex proportion of *Mora moro* by depth stratum derived from the surveys (1996–2019) in the Azores. The total number of individuals (n) refers to the total RPN (ind. 10^−3^ hooks). Red dashed line shows an equal sex ratio.

**Figure 7:**
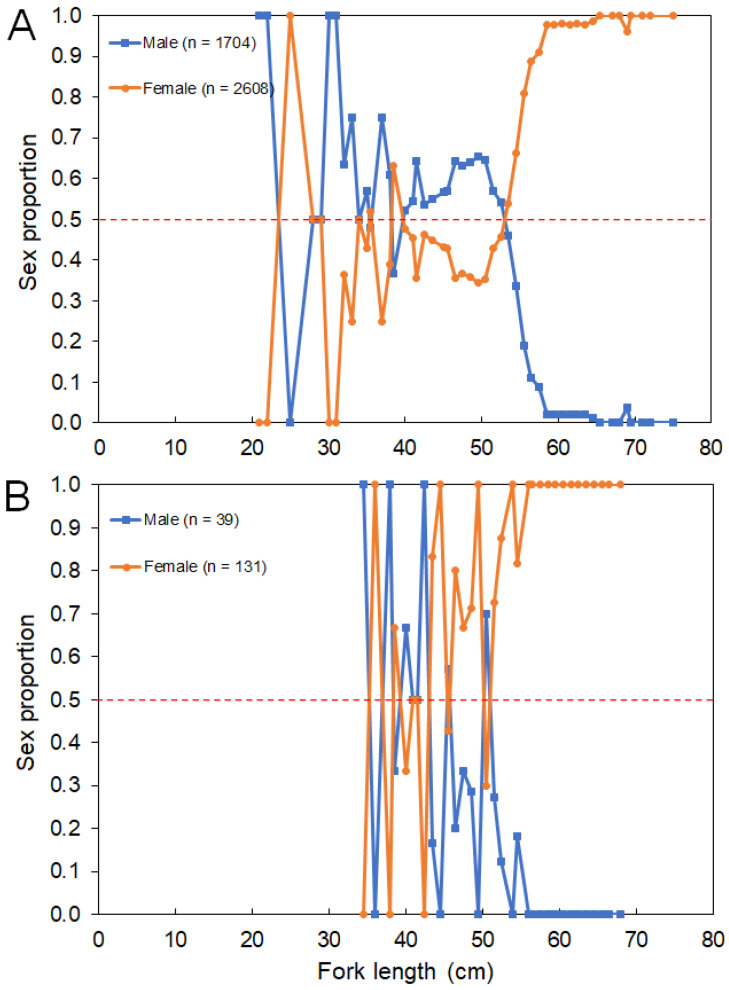
Sex proportion of *Mora moro* by fork length (*L_F_*)–class derived from the (**A**) surveys (1996–2019) and (**B**) commercial catches (2005–2017) in the Azores. Total number of individuals (n) refers to the total number of sampled *M. moro*. Red dashed line shows an equal sex ratio.

The overall sex ratios (M:F) observed from the survey and commercial catches were 0.66:1 and 0.30:1, respectively. No sexual segregation by area was observed, with females being the most abundant sex captured in both island (0.74:1) and seamount (0.49:1) regions. Females were significantly more abundant than males between 500 and 800 m (ꭓ^2^ > 4.258, *p* < 0.039) and between 1000 and 1200 m (ꭓ^2^ > 8.372, *p* < 0.004; Figure 6). No sex-related information by area or depth was available or reported in the fishery-dependent dataset. The sex ratio by size class from the survey and commercial catches showed females significantly dominating in *L_F_* larger than 55 cm (survey: ꭓ^2^ > 4.000, *p* < 0.045; fishery: ꭓ^2^ > 4.000, *p* < 0.045; Figure 7).

### 3.5. Reproduction

Thirty-nine males (34.5–55.0 cm *L_F_*) and 133 females (36.0–68.0 cm *L_F_*) from the DCF biological sampling program were analyzed. 

**Figure 8:**
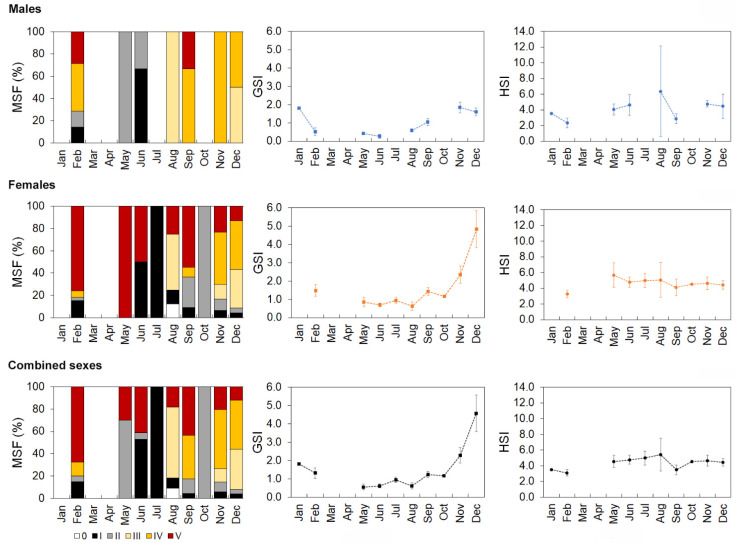
Monthly distribution of the maturity stage frequency (MSF), gonadosomatic index (GSI), and hepatosomatic index (HSI) for *Mora moro* analyzed from the commercial catches (2005–2017) in the Azores. 0—immature, I—resting, II—developing–beginning and the development of maturation, III—pre-spawning, IV—spawning–running, and V—spent. GSI and HSI are represented as mean value ± 0.95 confidence interval.

For males, a developing condition was found between February and June, pre-spawning occurred in August and December, and spawning from September to February, although data were lacking for January, March, April, July, and October. Males in the spent stage were present in February and September, and immature males were found in February and June (Figure 8). Females in the developing stage were present between September and February, pre-spawning occurred between August and December, and spawning occurred between September and February, although information was missing for January, March, and April. Females in the spent stage occurred from August to June and were in an immature condition between June and February (Figure 8). For combined sexes, a pattern similar to that observed for females was found, except for the developing stage, which occurred from September to June (Figure 8).

Statistically significant differences in the monthly mean GSI and HSI values of males (Welch’s test, GSI: *F* = 26.5, *p* < 0.001; HSI: *F* = 6.1, *p* = 0.016), females (Welch’s test, GSI: *F* = 19.1, *p* < 0.001; HSI: *F* = 3.44, *p* = 0.012), and both sexes combined (Welch’s test, GSI: *F* = 22.6, *p* < 0.001; HSI: *F* = 5.6, *p* < 0.001) were observed throughout the year. The GSI was higher from September to February for males (Bonferroni, *p* < 0.044), females (Bonferroni, *p* < 0.010), and both sexes (Bonferroni, *p* < 0.010; Figure 8). HSI variations were much less pronounced (Figure 8). Males presented higher values in November than in February and September (Bonferroni, *p* < 0.002). The female HSI was higher in June than in February (Bonferroni, *p* = 0.022). For both sexes, the mean HSI values registered in November and December were higher than in February (Bonferroni, *p* < 0.014).

The smallest mature male was observed at 38.0 cm *L_F_*, and the smallest mature female was observed at 38.5 cm *L_F_.* The logistic curve used to estimate the *L_50_* for both sexes showed a poor adjustment (R^2^ value = 0.03), so this result (*L_50_* = 17.3 cm *L_F_*, *β0* = −1.01, *β1* = 0.06) was not considered reliable.

### 3.6. Mortality, Exploitation Rate, and Size at Capture

Estimates of total mortality (*Z*) and natural mortality (*M*) for the period 2010–2016 were 0.21 year^−1^ and 0.16 year^−1^, respectively. The estimated fishing mortality of *F* = 0.05 corresponded to a low exploitation rate (*E* = 0.24). The *L_c_* was set at a 50.0 cm *L_F_*. Details on the mortality, exploitation rate, and size at capture estimates are available in Table 2 and Figure S2.

### 3.7. Catches and Landings

**Figure 9:**
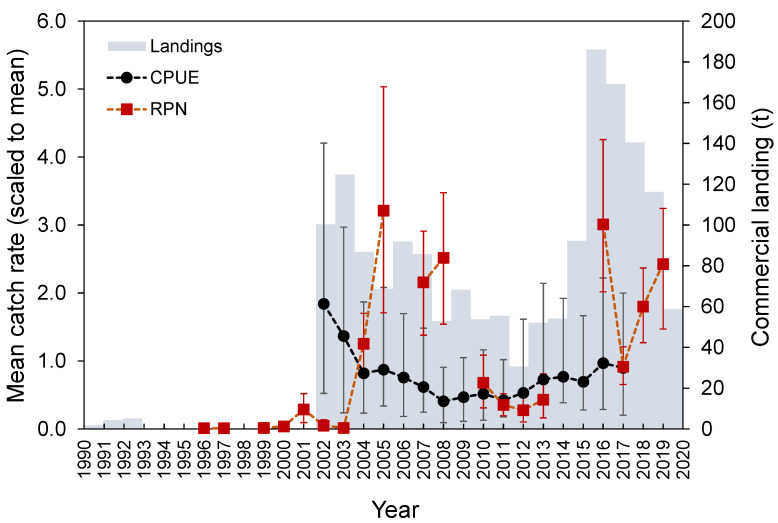
Official total landings (tons), mean (±0.95 confidence interval) CPUE (kg days at sea^−1^ vessel^−1^) from the commercial fishery and RPN (ind. 10^−3^ hooks) derived from the surveys catch rates of *Mora moro* in the Azores.

Marked interannual variability (Welch’s test, *F* = 10.4, *p* < 0.001; Figure 9) was observed in the survey-derived abundance index. *M. moro* was significantly less abundant between 2011 and 2013 when compared to 2005, 2008, and 2016 (Bonferroni, *p* < 0.038). The standardized CPUE from the commercial fleet showed an oscillation over time, with a peak in 2002, followed by a decreasing trend until 2008 and afterwards by some recovery (Figure 9). Landing time series analysis identified three main periods: one with zero-to-low values from 1990 to 2001, one with high values but with a decreasing trend between 2002 and 2012, and one with an increasing pattern and the highest landings values registered in the time series from 2013 onwards (Figure 9). A gradual decline was observed from 2016 to 2020. Overall, fishery-dependent and survey catches as well as commercial landings showed a bimodal pattern with peaks during the periods 2002–2005 and 2016–2020.

### 3.8. Fishing Effort

**Figure 10:**
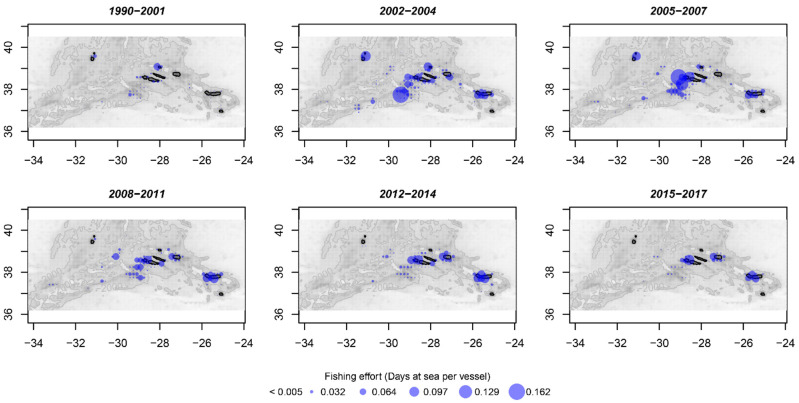
Spatial distribution of average fishing effort (days at sea per vessel) of the Azorean fishing fleet in 1990–2017. Gray scale indicates the bathymetry (seafloor depth) from shallow (dark gray) to deep (white) waters. Black color represents a land mask. Blue bubbles represent the proportional fishing effort. Data from the EU Data Collection Framework (DCF) port inquiries.

Areas adjacent to the islands represented on average the most important grounds in terms of fishing effort of the Azorean fleet (Figure 10). A strong intensification of fishing effort on seamounts at the south of the central islands group was noted between 2002 and 2007 (Figure 10). After this period, an important part of the fishing effort was displaced from shore to offshore seamounts, namely on the MAR (Figure 10).

## 4. Discussion

*Mora moro* has been registered throughout the Azores on mud, sand, and rock bottoms at depths below 300 m. An increase in the occurrence and abundance of this species at depths between 800 and 1000 m reflected a spatial distribution restricted to seamounts, offshore banks, and island slopes, in which this depth range is mostly available. Variations in demersal fish abundance and distribution have frequently been associated with depth [48] or depth-related environmental factors (e.g., water temperature, oxygen saturation, and salinity) [49,50]. The inclusion of these physicochemical variables might improve habitat predictions, but they were not available at a fine-scale resolution and when derived from global data sets, may show low predictive power [51].

Small to large individuals were observed around islands and seamount areas of the Azores. The fishing gear (hook and line) may not have been effective at sampling smaller individuals (few specimens under 35 cm were caught in this study; Figure 3), but even so, a larger–deeper trend was observed, as previously reported for the Mediterranean [52]. The presence of smaller individuals in shallower waters (mainly up to 650 m) has been related to oceanographic processes that favor the concentration of decapod crustacean species at the seabed (at a depth of approximately 400–600 m), providing *M. moro* post-larvae and juveniles with an enriched food supply [52]. However, the applicability of this hypothesis to the NE Atlantic needs to be investigated. Similar depth–size trends have been reported for other demersal and deep-water fish species, such as the blackbelly rosefish *Helicolenus dactylopterus* [53], Kaup’s arrowtooth eel *Synaphobranchus kaupi* [54], and the greater forkbeard *Phycis blennoides* [55]. Although the larger–deeper distribution pattern is a common tendency in deep-sea fish assemblages, it cannot be considered a general rule [56,57]. 

Growth parameters were consistent with those in the literature [19] and indicated that *M. moro* is a long-living and slow-growing fish. Females showed an increase in dominance with an increasing size such as in the Mediterranean [58] and Pacific [59]. The potential size of sexual inversion at a 55 cm *L_F_*, at which almost all individuals were females (Figure 7), could be considered a signal of sequential hermaphroditism. However, this reproductive strategy was not confirmed by the macroscopic observation of the gonads. In fact, growth rates estimated by sectioned otolith readings for males and females caught in the Azores were different, with females reaching a larger size and growing slower than males [19]. Sectioned otoliths provided ages between 9 and 59 years for females with a 28–76 cm *L_F_*, and males were aged between 8 and 45 years with a 22–66 cm *L_F_* [19]. This sexual growth dimorphism was also observed for other morid fishes, such as the blue antimora *Antimora rostrata* [60].

Based on our findings, the reproductive season for both sexes in the Azores region takes place from August to February (the time between the appearance of the first individual during pre-spawning and the last during spawning), with a spawning peak in November and December (when individuals in the spawning condition were detected). This period, in addition to being corroborated by the GSI and HSI results, was similar to that previously suggested in the Azores [19] and Mediterranean [6,52,58]. As individuals were reproductively active at the same time of the year in different geographic areas, the probability of mixing between the Azorean and Mediterranean populations can be considered reduced. However, fertilized eggs float to the mixed layer or shallower waters, and fish larvae develop in the surface water layer [52]. Therefore, connectivity may occur during the early life-history stages (eggs and larvae) since long-distance migrations of adults are not consistent with the sedentary behavior of this species [61,62]. Further studies are fundamental to model larval transport and investigate pathways across the Mediterranean Sea to the NE Atlantic. The smallest sizes of mature males and females were larger than those observed in the Mediterranean area. The smallest sizes estimated for *M. moro* in the Balearic Sea (the Western Mediterranean) were a 32 cm total length (*L_T_*) (or a 29 cm *L_F_*) for males and a 34 cm *L_T_* (a 31 cm *L_F_*) for females [58]. Estimates of *L_50_* were skewed mainly due to a poor data series with large size classes (Figure 3). The possibility of significant macroscopic misclassification of maturing or resting fish as immature may also have influenced it in some way. Though gonad classification is considered by samplers to be easily achieved without microscopic confirmation, it is increasingly recommended to validate the macroscopic staging through histology [63]. 

Annual trends in abundance indices, catch, and mean size in the catch were marked by changes in the Azorean fishing fleet operational behavior. Traditionally, the Azorean fleet targeting on demersal fishes operates between 200 and 600 m [64]. Therefore, *M. moro* with medium sizes occupying depths down to 600 m constitutes the fraction of the population most vulnerable to capture. In the Azores, the exploitation phase of this resource started in 2002, with a high fishing effort around the islands and seamounts closest to the coast. Because of this intensive fishing, catches and abundances decreased in the subsequent period (2008–2014). An increase in the mean *L_F_* in the catch was also observed during 2008–2014, and it may have been caused by larger individuals that became more available for fishing after a decrease in the abundance of medium-sized fishes. With the reorientation of the fleet to the MAR due to the implementation of fishing area restrictions [5,48], the relief in the fishing effort at the upper fringe of coastal habitats of the species allowed the populations to rebuild themselves to higher levels (see Figure 9 and Figure 10). The exploitation rate (*E*) estimated for the most recent 2010–2016 period was below the optimum level of 0.5, and the fishing mortality (*F*) was lower than natural mortality (*M*), indicating that this species is currently not overexploited [43]. Recent studies in seamount areas have shown that the impacts of fishing on this stock are no longer pronounced because there is a fraction of the population that is not exploited and would be able to restore the levels of exploited stock by recruitment [48]. 

As the commercial interest in this species is increasing, as well as offshore deep-water efforts, improving data quality and input information for stock assessment should be a priority. In this sense, additional contributions have been provided through empirical estimations of relevant biological and fishery parameters, such as length at first maturity (*L_m_*), length at maximum possible yield (*L_opt_*), life span (*t_max_*), and theoretical age at length zero (*t*_0_), using the estimated growth parameters (*L_∞_* and *k*; Table 2) [65,66]. Like other deep-water species [18,53,67,68,69,70], *M. moro* can be considered vulnerable to overfishing because of its large size, slow growth, low natural mortality (Table 2), long life span, and late maturity (Table S1). However, besides the observed low *E* and *F* (Table 2), the empirical equations suggested an apparently healthy fished population in the Azores, with the length at which 100% of individuals are vulnerable to capture (*L_c_*; Table 2) above the *L_m_* and *L_opt_* (Table S1), and the mean *L_F_* in the catch (Figure 5) above the *L_c_*, *L_m_*, and *L_opt_*. It is important to highlight the estimated empirical values are preliminary and should be interpreted with caution. These results must first be validated (e.g., [71,72]). Only then can this information be used for management until specific data become available.

## 5. Conclusions

This study unravels and highlights important and crucial aspects for managing the *M. moro* resource. The stock structure indicated that the population inhabiting the ICES Subarea 10a2 may be considered a discrete FMU. The main evidence for the self-sustaining population assumption consists in their observed sedentary behavior, their occurrence in the region of small and immature to large and reproductively active individuals, and signals of their spawning ground. No information on *M. moro* populations inhabiting other NE Atlantic regions is currently available. However, tagging studies using parasites as biological tags support the heterogeneity among populations from nearby geographic areas [62]. Thus, following the precautionary principle, it is recommended that exploratory stock assessment analyses be carried out under this assessment unit assumption. Meanwhile, further studies (e.g., larval ecology, reproduction, genetics, tagging, morphometry, and biological and physical–chemical interactions) should be performed to generate adequate data and make meaningful progress in stock characterization and metapopulation modeling. While our results showed no signal of overexploitation of the *M. moro* stock in the Azores, deep-water fishing restrictions may become critical if fishing pressure on mega-spawners is increased. At the same time, if a fishing season closure is required, then the period between November and December should be taken into account. Severe fishing pressures during this spawning period may affect the reproductive success of the species, reducing the number of spawners and subsequently the number of recruits. The next steps should involve assessing the stock size and biological reference points using data-limited methods to produce the maximum sustainable yield or a proxy.

## Figures and Tables

**Table 1:** Detailed summary table of generalized additive model (GAM) results for the *Mora moro* abundances derived from the surveys (1996–2019) in the Azores. Substrate type: coarse sediment (C. Sed), mixed sediment (Mix.Sed), mud (Mud), muddy sand (Mud.S), rock (Rock), sand (Sand), and sandy mud (Sand.M).

Family	Link Function		Formula				Adjusted R^2^		Deviance Explained
Binomial	logit		RPN.Bi ~ s (Longitude, Latitude) + s (Depth, k = 4) + Substrate				0.442		45.40%
Gaussian	identity		RPN ~ s (Longitude, Latitude) + s (Depth, k = 4) + Substrate				0.223		23.90%
**Binomial**					**Gaussian**				
**Parametric coefficients**									
	**Estimate**	**Std. Error**	**z Value**	**Pr(>|z|)**		**Estimate**	**Std. Error**	**z Value**	**Pr(>|z|)**
(Intercept)	−3.131	0.219	−14.329	<0.001	(Intercept)	3.301	0.820	4.028	<0.001
SubstrateMix.Sed	0.195	0.210	0.928	0.354	SubstrateMix.Sed	0.718	0.855	0.840	0.401
SubstrateMud	0.384	0.289	1.331	0.183	SubstrateMud	0.695	1.062	0.654	0.513
SubstrateMud.S	0.425	0.368	1.154	0.248	SubstrateMud.S	0.334	1.379	0.242	0.809
SubstrateRock	0.223	0.259	0.858	0.391	SubstrateRock	0.818	1.078	0.759	0.448
SubstrateSand	0.211	0.221	0.955	0.340	SubstrateSand	0.633	0.876	0.722	0.470
SubstrateSand.M	−1.199	0.583	−2.059	0.040	SubstrateSand.M	−0.496	2.693	−0.184	0.854
**Approximate Significance of Smooth Terms**									
	**edf**	**Ref. df**	**Chi. sq**	***p*-Value**		**edf**	**Ref. df**	**Chi. sq**	***p*-Value**
s (Longitude, Latitude)	24.430	27.530	74.540	<0.001	s (Longitude, Latitude)	27.832	28.893	16.840	<0.001
s (Depth)	2.990	3.000	1610.380	<0.001	s (Depth)	2.866	2.987	25.990	<0.001

**Table 2:** Growth and fishery parameters for *Mora moro* in the Azores estimated from *L_F_*–frequency data for the period 2010–2016. Data from the EU Data Collection Framework (DCF). Lower and upper denote (^a^) 95% confidence interval, (^b^) standard deviation, or (^c^) standard error limits of the estimates.

Parameters	Method	Estimates	Lower	Upper
Asymptotic length (*L_∞_*; cm *L_F_*)	ELEFAN_GA_Boot [35]	77.68	75.17 ^a^	79.61 ^a^
Growth coefficient (*k*; year^−1^)	ELEFAN_GA_Boot [35]	0.07	0.05 ^a^	0.08 ^a^
Growth performance index (*⏀*)	ELEFAN_GA_Boot [35]	2.63	2.54 ^a^	2.71^a^
Natural mortality (*M*; year^−1^)	[42]	0.16	−0.03 ^b^	0.35 ^b^
Total mortality (*Z*; year^−1^)	[41]	0.21	0.20 ^c^	0.22 ^c^
Fishing mortality (*F*; year^−1^)	[42]	0.05	0.03 ^b^	0.07 ^b^
Exploitation rate (*E*)	[43]	0.24		
Catchability coefficient (*q*)	[42]	0.03	−0.08 ^b^	0.14 ^b^
Length of full selectivity (*L_c_*; cm *L_F_*)	[41]	50.0		

## Data Availability

Data supporting reported results are available from the corresponding author upon reasonable request.

## Supplementary Materials

The following are available online at https://www.mdpi.com/article/10.3390/biology10060522/s1, Figure S1. Growth curves (dashed lines) for *Mora moro* in the Azores plotted through the *L_F_*–frequency data obtained using bootstrapped ELEFAN_GA model. Black bars indicate positive values (peaks), whereas white bars indicate negative peaks. Shading refers to the difference between moving averages. Data from the EU Data Collection Framework (DCF) for the period 2010–2016. Figure S2. Estimates of annual total mortality (*Z*) and fishing mortality (*F*) rates for *Mora moro* in the Azores. Data from the EU Data Collection Framework (DCF) for the period 2010–2016. Table S1. Estimates of biological and fishery parameters for *Mora moro* calculated from the empirical relationships between the length at first maturity (*L_m_*), length at maximum possible yield (*L_opt_*), life span (*t_max_*), theoretical age at length zero (*t_0_*), and the asymptotic length (*L_∞_*) and growth coefficient (*k*). The values of *L_∞_* and *k* derived from the *L_F_*–frequency data collected for the period 2010–2016 as part of the EU Data Collection Framework (DCF).
