# Supplementary material for: Contributions to Management Strategies in the NE Atlantic Regarding the Life History and Population Structure of a Key Deep-Sea Fish (Mora Moro)
Source: Biology (Basel). 2021 Jun 11;10(6):522. doi: 10.3390/biology10060522 (PMC8230854; doi:10.3390/biology10060522)
Supplement: Supplementary file 1 [file biology-10-00522-s001.zip › biology-1189051-supplementary.pdf]

Contributions to Management Strategies in the NE Atlantic Regarding the Life History and Population Structure of a Key Deep-Sea Fish (*Mora Moro*)

Régis Santos [1,2,*], Wendell Medeiros-Leal [1,2], Osman Crespo [2], Ana Novoa-Pabon [2,3] and Mário Pinho [1,2,3]

[1] IMAR Institute of Marine Research, University of the Azores, 9901-862 Horta, Portugal; wendell.mm.silva@uac.pt (W.M.-L.); mario.rr.pinho@uac.pt (M.P.)
[2] Okeanos R&D Centre, University of the Azores, 9901-862 Horta, Portugal; osman.crespo@gmail.com (O.C.); 19anita89@gmail.com (A.N.-P.)
[3] Department Oceanography and Fisheries, Faculty of Science and Technology, University of the Azores, 9901-862 Horta, Portugal
* Correspondence: regisvinicius@gmail.com; Tel.: +351-935098870

Supplementary material

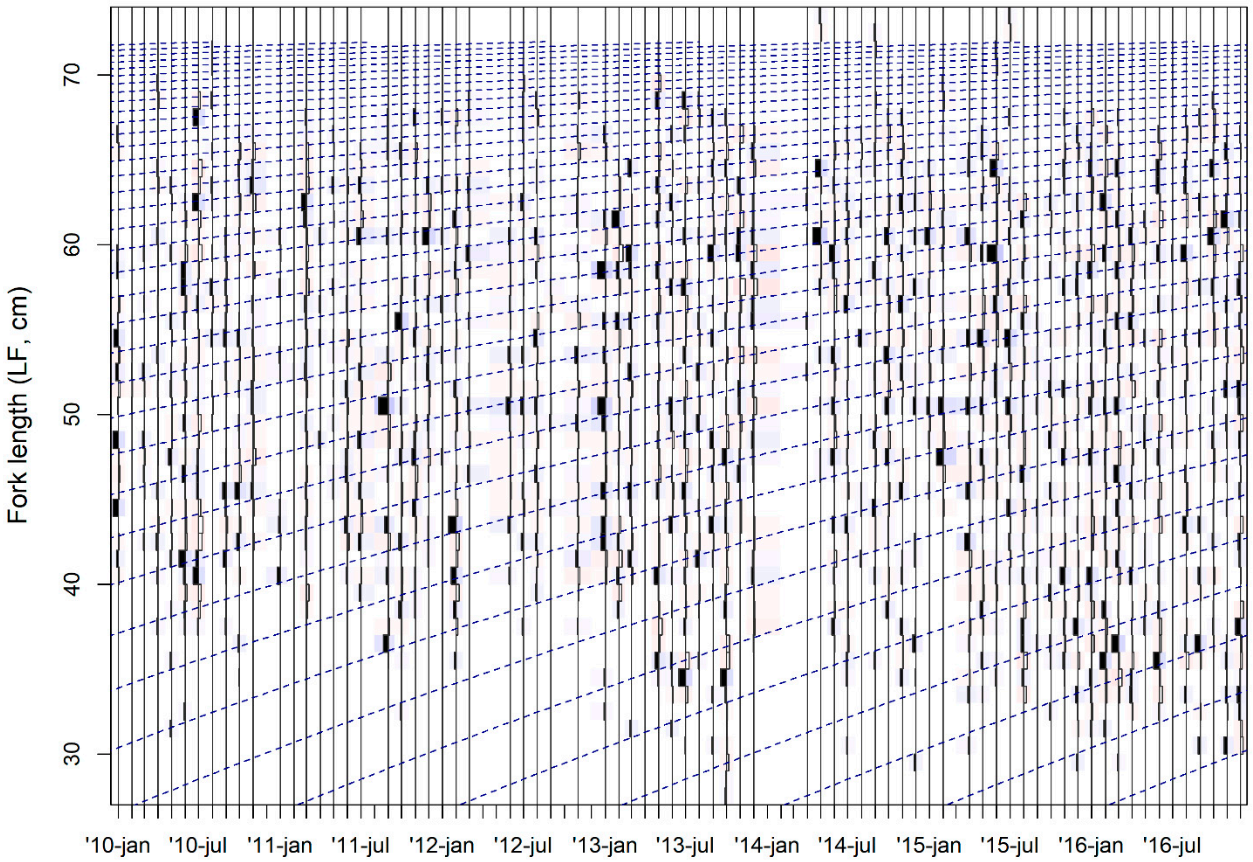

Figure S1. Growth curves (dashed lines) for *Mora moro* in the Azores plotted through the L_F–frequency data obtained using bootstrapped ELEFAN_GA model. Black bars indicate positive values (peaks), whereas white bars indicate negative peaks. Shading refers to the difference between the moving averages. Data from the EU Data Collection Framework (DCF) for the period 2010–2016.

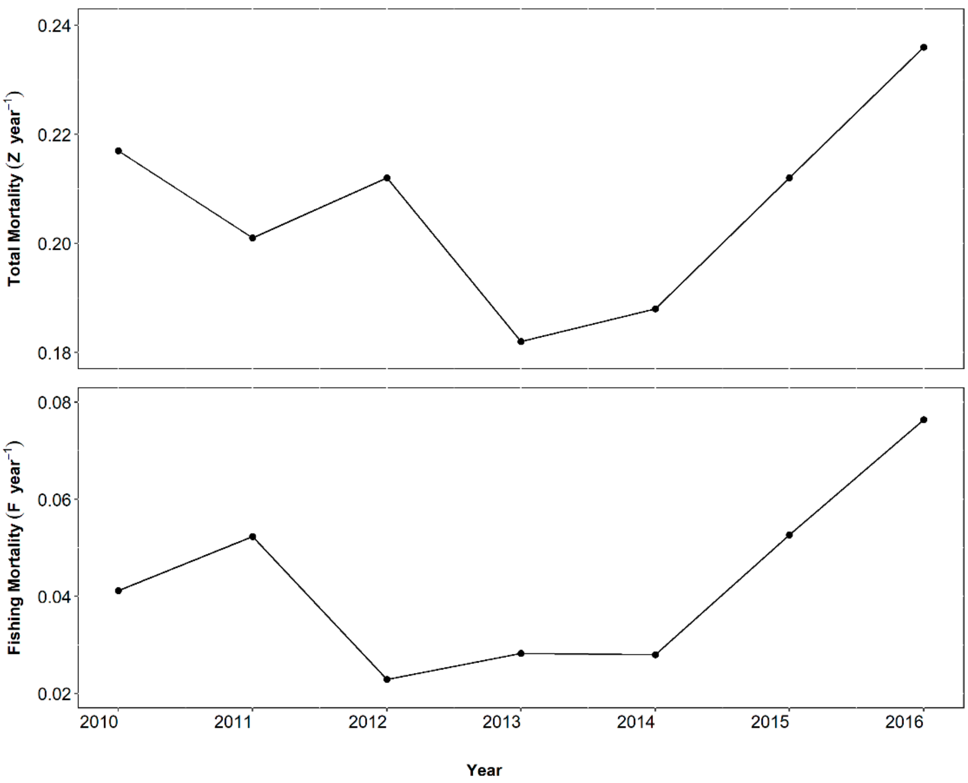

Figure S2. Estimates of annual total mortality (*Z*) and fishing mortality (*F*) rates for *Mora moro* in the Azores. Data from the EU Data Collection Framework (DCF) for the period 2010–2016.

Table S1. Estimates of biological and fishery parameters for *Mora moro* calculated from the empirical relationships between the length at first maturity (L_m), length at maximum possible yield (L_{opt}), life span (t_{max}), and theoretical age at length zero (t_0), and the asymptotic length (L_∞) and growth coefficient (k). The values of L_∞ and k derived from the L_F–frequency data collected for the period 2010–2016 as part of the EU Data Collection Framework (DCF).

Parameter	Estimates	Empirical formula	Reference
Length at first maturity (L_m)	41.60 cm L_F	$\log(L_m) = 0.8979 \times \log(L_\infty) - 0.0782$	[1]
Length at maximum possible yield (L_{opt})	49.62 cm L_F	$\log(L_{opt}) = 1.053 \times \log(L_m) - 0.0565$	[1]
Life span (t_{max})	42.86 years	$t_{max} = 3 / k$	[1]
Theoretical age at length zero (t_0)	0.52 years	$\log(-t_0) = -0.3922 - 0.2752 \times \log(L_\infty) - 1.038 \times \log(k)$	[2]

References

1. Froese, R.; Binohlan, C. Empirical relationships to estimate asymptotic length, length at first maturity and length at maximum yield per recruit in fishes, with a simple method to evaluate length frequency data. *J. Fish Biol.* **2000**, *56*, 758–773, doi:https://doi.org/10.1111/j.1095-8649.2000.tb00870.x.

2. Pauly, D. *Theory and Management of Tropical Multi-Species Stocks: A Review, with Emphasis on the Southeast Asian Demersal Fisheries.*; International Center for Living Aquatic Resources Management: Manila, Philippines, 1979; Vol. 1;.
